# A novel nitidine chloride nanoparticle overcomes the stemness of CD133^+^EPCAM^+^ Huh7 hepatocellular carcinoma cells for liver cancer therapy

**DOI:** 10.1186/s40360-022-00589-z

**Published:** 2022-07-12

**Authors:** Danni Li, Qiying Zhang, Yuzhu Zhou, Hua Zhu, Tong Li, Fangkai Du

**Affiliations:** 1 School of Chemistry and Chemical Eengineering, Guangxi Minzu University, No.158, Da Xue Xi street, Xixiangtang District, Nanning, 530006 Guangxi Province China; 2 College of Pharmacy, Guangxi University for Chinese Medicine, No.13 , Wu He street, Qingxiu District, Nanning, 530200 Guangxi Province China

**Keywords:** Nitidine chloride nanoparticles, EpCAM^+^ /CD133^+^ Huh7 cells, AQP3/STAT3/CD133 pathway, Huh7 cells xenograft nude mice

## Abstract

**Background:**

Stemness of CD133^+^EPCAM^+^ hepatocellular carcinoma cells ensures cancer resistance to apoptosis,which is a challenge to current liver cancer treatments. In this study, we evaluated the tumorcidal activity of a novel nanoparticle of nitidine chloride (TPGS-FA/NC, TPGS-FA: folic acid modified D-α-tocopheryl polyethylene glycol 1000 succinate, NC: nitidine chloride), against human hepatocellular carcinoma (HCC) cell line Huh7 growth in vitro and in vivo.

**Methods:**

Huh7 cells were treated with TPGS-FA/NC. Cell proliferation was assessed using MTT and colony assays. The expression of cell markers and signaling proteins was detected using western blot analyses. A sphere culture technique was used to enrich cancer stem cells (CSC) in Huh7 cells. TPGS-FA/NC (7.5, 15, 30, 60, 120 μg/mL) dose-dependently inhibited the proliferation of HCC cells, which associated with a reduction in AQP3 and STAT3 expression. Importantly,TPGS-FA/NC (10, 20, and 40 μg/mL) significantly reduced the EpCAM^+^/CD133^+^cell numbers, suppressed the sphere formation. The in vivo antitumor efficacy of TPGS-FA/NC was proved in Huh7 cell xenograft model in BALB/c nude mice, which were administered TPGS-FA/NC(4 mg· kg − 1· d − 1, ig) for 2 weeks.

**Results:**

TPGS-FA/NC dose-dependently suppressed the AQP3/STAT3/CD133 axis in Huh7 cells. In Huh7 xenograft bearing nude mice, TPGS-FA/NC administration markedly inhibited Huh7 xenograft tumor growth .

**Conclusions:**

TPGS-FA/NC inhibit HCC tumor growth through multiple mechanisms, and it may be a promising candidate drug for the clinical therapy of hepatocellular carcinoma.

**Supplementary Information:**

The online version contains supplementary material available at 10.1186/s40360-022-00589-z.

## Background

Multiple drugs have been used broadly in liver cancer therapy, but their water-insolubility and toxicity have raised serious concerns [1, 2]. Nitidine chloride has been developed in the past two decades due to its promise pharmacological action. However, Nitidine chloride have limited applications because of potential organ damage, hypersensitivity, and neurotoxicity [3, 4]. Thus, facing the liver cancer therapy challenge, it is urgent to discover promising drug target and methods to achieve safe and effective tumor inhibition.

The cancer stem cells(CSC) are identified as stem cell properties,which revealed the existence of CSC in HCC [5, 6]. Intriguingly, CD133^+^EpCAM^+^phenotype precisely represented the characteristics of CSC in Huh7 cells [7–11]. Currently some chemotherapeutic drugs primarily inhibit the growth of differentiated tumor cells with no impact on CSC [12, 13]. Cancer stem cells (CSCs) maintain the stemness to ensure their survival and growth, and becoming resistant to current treatments [14–16]. The intrinsic pathway of CD133^+^Huh7 cells is regulated by the AQP3 protein in the progression and metastasis of several malignant tumors [17–20]. Furthermore, Nek2 is the critical regulator of the centrosome, making hepatocellular carcinoma more resistance to current treatments [21, 22]. In this regard, functional AQP3 and Nek2 is mutated or highly expressed in hepatocellular carcinoma, these molecular and cellular mechanisms may be overcome by the pharmacological action of AQP3/STAT3/CD133 pathway degradation and Nek2 inhibition. Thus, in this study, we comprehensively investigated the role of TPGS-FA/NC in the antitumor effect and explore its mechanisms via AQP3/STAT3/CD133 axis, which would offer therapeutic strategies against liver cancer.

## Methods

### Materials

TPGS-FA/NC was synthesized in our laboratory and dissolved in DMSO. DMEM was purchased from Life Technologies (AB & Invitrogen)(Gibco, Suzhou, China). Fetal bovine serum (FBS) was purchased from Gemini (Gemini Calabasas, CA, USA). Huh7 cells were purchased from Procell Life Science & Technology Co. Ltd. on July 11, 2019 (Wuhani, China). L-02 cells were purchased from Procell Life Science & Technology Co. Ltd. on January 18, 2019 (Wuhani, China). Recombinant human bFGF (bFGF), recombinant human 1epidermal growth factor (EGF) and MTT were purchased from Beijing Solarbio Science & Technology Co., Ltd.(Solarbio,Beijing, China). B27 (× 50) were purchased from Thermofish Scientific(Thermofish,waltham, USA). DMEM/F-12, insulin-Transferrin-Selenium (ITS× 100), L-glutamine (× 100) were purchased from Procell Science&Technology Co.,Ltd.(Procell,Wuhan, China). Anti-CD133 (AC133)-phycoerythrin (PE) and anti-CD326 (EpCAM)-allophycocyanin (APC) antibodies and isotype-matched mouse anti-IgG1-PE and anti-IgG1-APC were purchased from MiltenyiBiotec (North Rhine-Westphalia, Germany). Anti-phosphoSTAT3 (Tyr705), STAT3, JAK1, JAK2, AQP3, EpCAM, NEK2 were purchased from the Beijing Solarbio Science & Technology Co., Ltd. (Solarbio,Beijing, China). Anti-CD133 and anti-GAPDH were purchased from the Proteintech Science&Technology Co.,Ltd.(Proteintech, suzhou, China). Anti-rabbit secondary antibodies were purchased from Thermo Fisher Scientific Science&Technology Co.,Ltd.(Thermo Fisher Scientific, Shanghai, China). DAPI was obtained from Shanghai Beyotime Biotechnology Co. Ltd. (Beyotime,Shanghai, China). The iFluor TM 647 phalloidin iFluor™ were purchased from Yeasen Biotechnology Co., Ltd. (Yeasen,Shanghai, China). 5-fluorouracil (5-Fu) was purchased from MedChemExpress (MCE, Monmouth Junction, NJ, USA).

### Cell culture

Huh7 cells and normal hepatic cell line L-02 were cultured in DMEM 10% FBS containing 10% FBS, 100 U/mL penicillin, and 50 mg/mL streptomycin at 37 °C in a humidified 5% CO2 incubator.

### Tumor sphere formation assay and flow cytometric analysis

Primary sphere cells were obtained by culturing Huh7 cells in sphere-forming conditioned DMEM/F12, supplemented with FGF (20 ng/mL), EGF (20 ng/mL), B27 (1×), and L-glutamine (1×) in 6-well ultra-low attachment plates The primary sphere cells (1 × 10^3^ cells/well) were incubated with or without TPGS-FA/NC for 7d. The second and third passages of the cells were grown for 7 d in the absence of TPGS-FA/NC. To examine TPGS-FA/NC effects on the subpopulation of cells that expressed EpCAM and CD133, cells were incubated with anti-AC133-PE and anti-EpCAM-APC antibodies and analyzed by flow cytometry. Isotype-matched mouse anti-IgG1-PE and anti-IgG1-APC were used as controls.

### Confocal microscopy imaging

Huh7 cells cells were seeded on glass cover-slips and cultured at 37 °C overnight. Rhodamine B isothiocyanate 540 labeled TPGS-FA/NC were incubated with cells at a final concentration of 100 nM for 4 h at 37 °C. After washing twice with PBS buffer, cells were fixed with 4% formaldehyde and washed again, followed by treatment with 0.1% Triton X-100 in PBS buffer for 5 min and subsequent cytoskeleton staining with iFluor TM 647 phalloidin iFluor™ for 30 min at room temperature. Containing DAPI for cell nucleus staining and assayed on Leica SP8 confocal microscope (Leica Corp.).

### Western blotting

Cells were lysed in RIPA lysis buffer with PMSF and protease in-hibitors. Total protein lysates were boiled with loading sample buffer containing 8% SDS-PAGE. Separated proteins were transferred onto PVDF membranes. PVDF membrane blots were blocked in 10% skimmed milk for 0.5-1 h at room temperature, washed in Tris-buffered saline with Tween 20 (TBS-T) and incubated overnight at 4 °C with rabbit anti-phosphoSTAT3 (Tyr705), anti-STAT3, anti-JAK1, anti-JAK2, anti-AQP3 CD33, anti-GAPDH. Anti-rabbit IgG was used as the second antibody.

### Immunohistochemistry (IHC)

AQP3/CD133/EPCAM/NEK2 expression was analyzed in paraffin-embedded specimens obtained from nude mice tumor tissue. Tissue sections were incubated with anti-AQP3 (1:100, Solarbio), anti-CD133 (1:100, Solarbio), anti-EPCAM (1:100, Solarbio), and anti-NEK2 (1:100, Solarbio) overnight at 4 °C. Then, the sections were incubated with biotinylated goat anti-rabbit IgG as a secondary antibody (Zhongshan Kit, China) for 30 min at 37 °C. The specimens were assessed three times.

### In vivo biodistribution assay

Rhodamine B isothiocyanate labeled TPGS-FA/NC (2 mg.kg^− 1^, NC per body weight) were systemically administered via the tail vein into Huh7 tumor bearing mice. PBS-injected mice were used as fluorescence negative controls. The whole-body imaging of mice was conducted at 8 h using an IVIS system (XMRS) with excitation at 535 nm and emission at 694 nm. The mice were sacrificed at 8 h post-injection by the inhalation of CO_2_ followed by cervical dislocation, and major organs were collected and subjected to fluorescence imaging for the assessment of biodistribution profiles. The fluorescence imaging datas of average radiant efficiency ([ps^− 1^cm^− 2^sr^− 1^] [μWcm^− 2^] ^− 1^) were quantitative by IVIS system (XMRS) program.

### Magnetic-activated cell sorting assay

Determine cell number,Centrifuge cell suspension at 300×g for 10 minutes, Aspirate supernatant completely. Resuspend cell pellet in 300 μL of buffer per 5 × 10^7^ total cells. Add 100 μL of FcR Blocking Reagent per 5 × 10^7^ total cells and mix well. Add 100 μL of EpCAM microbeads per 5 × 10^7^ total cells. Mix well and incubate for 30 minutes (2 − 8 °C). Wash cells by adding 5 − 10 mL of buffer per 5 × 10^7^ cells and centrifuge at 300×g for 10 minutes. Aspirate supernatant completely and suspend up to 10^6^ cells in 500 μL buffer, proceed to magnetic separation, EpCAM Huh7 cells were collected. Followed above methods, EpCAM Huh7 cells were sorted after CD133 microbeads incubation. EpCAM^+^and CD133^+^Huh7 cells were collected by magnetic separation.

### In vivo tumor inhibition by TPGS-FA/NC nanoparticles

Freshly sorted EpCAM^+^ CD133^+^ Huh7 cells were collected in sterile DMEM without FBS. 1 × 10^7^ Huh7 cells/site cell suspension, mixed with matrigel (BD Biosciences, CA) (1:1), was subcutaneously injected into the axillary of nude mice, which were randomly divided into four groups (*n* = 5 biologically independent animals). When the tumor nodules had reached a volume of 75 mm^3^, the nude mouse were used for tumor inhibition studies. Samples were administrated by i.v. injection in a total of 5 doses (4 mg kg^− 1^, NC per body weight) every other day. Tumor volume, calculated as (length×width^2^)/2, and mouse weight were monitored every other day. Data were statistically analyzed by two-tailed unpaired t-test and presented as mean ± SD; *p < 0.05; **p < 0.01; ***p < 0.001.

#### Statistics

Statistical differences were evaluated using two-tailed unpaired t-test with GraphPad software, and statistically significant differences are denoted as *p < 0.05, **p < 0.01, and ***p < 0.001. No adjustments were made for multiple comparisons.

## Results

### TPGS-FA/NC inhibited cell proliferation and targeted the Huh7 cells

**Fig. 1:**
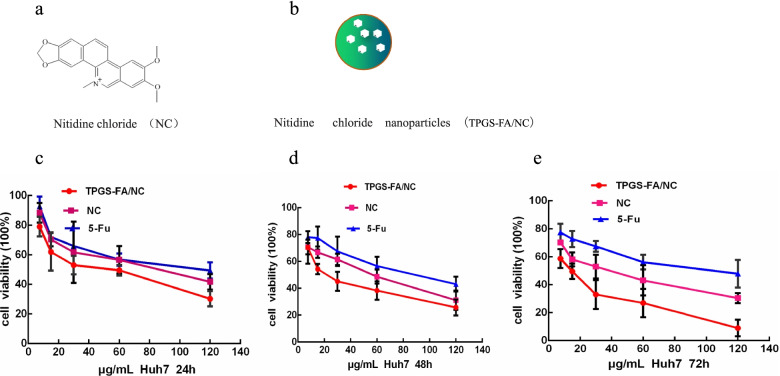
The effect of TPGS-FA/NC on Huh7 cell proliferation (**a**) Chemical structure of NC (**b**) TPGS-FA/NC nanoparticles. **c-e** In vitro cytotoxicity study of TPGS-FA/NC by MTT assay in 24, 48 and 72 h. (Data are presented as mean ± SD *n* = 3 independent samples)

**Fig. 2:**
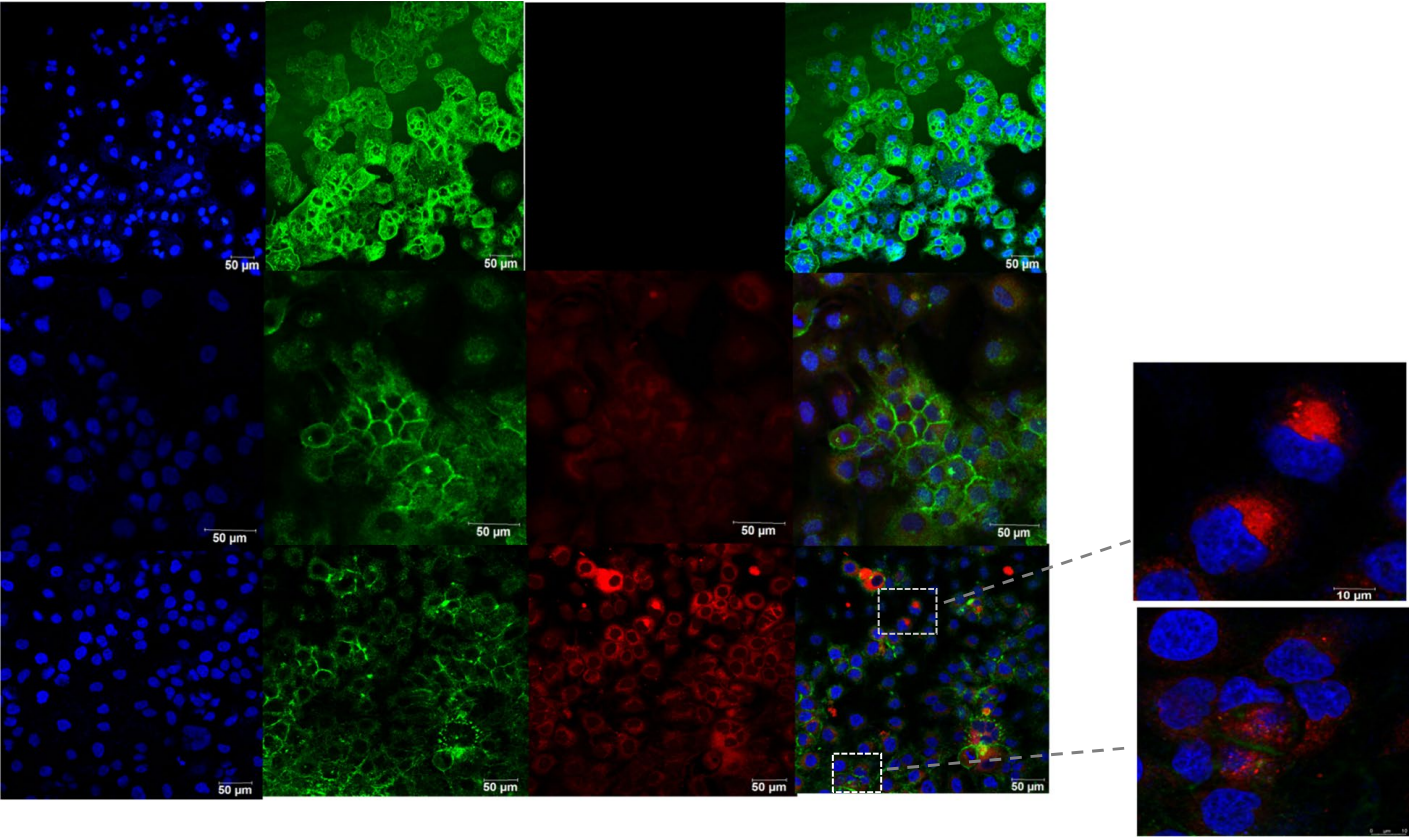
In vitro Huh7 cells binding of TPGS-FA/NC nanoparticles. Fluorescence staining of Huh7 cells, which were treated withTPGS-FA/NC for 48 h, was visualized by fconfocal microscopy. (blue: nucleus; green: cytoskeleton; red: TPGS-FA/NC nanoparticles. Scale bar: 50 μm for original images, and 10 μm for magnified image)

We found that Huh7 cells (2 × 10^3^cells/well) were seeded into 96-well plates and treated with TPGS-FA/NC (0–120 μg/mL) for 24, 48, and 72 h (Fig. 1). Cell proliferation was assessed using MTT in a concentration- and time-dependent manner. To evaluate nanoparticles targeting tumor capability, the RhodamineB isothiocyanate 540 fluorophore was attached to TPGS-FA. Confocal microscope imaging showed that TPGS-FA/NC nanoparticles entered the Huh7 cells in vitro, compared with the control groups (Fig. 2).

### TPGS-FA/NC reduced hepatic cancer stem-like cells

**Fig. 3:**
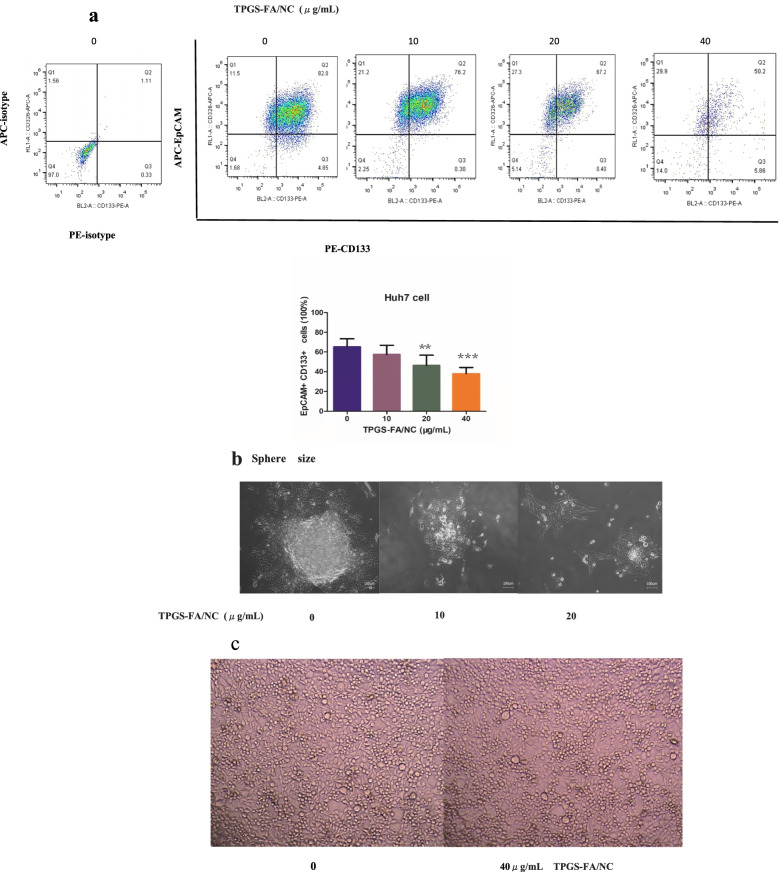
The effect of TPGS-FA/NC on hepatic cancer stem-like cells. **a** TPGS-FA/NC reduced the population of EpCAM+/CD133+ cells for 48 h. Data are presented as mean ± SD and **p < 0.01 and ***p < 0.001. **b** TPGS-FA/NC reduced the sizes Huh7 primary spheres (magnification,× 400). **c** Phase contrast photomicrographs obtained by normal hepatocyte cell L-02 treated with TPGS-FA/NC at a concentration of 40 μg/mLfor 48 h (magnification,× 100)

To investigate whether TPGS-FA/NC suppressed hepatic CSCs, we enriched the hepatic CSC populations in the Huh7 cell lines using the sphere culture technique. The flow cytometric analysis demonstrated that the EpCAM+/CD133+ cells accounted for 82.0% of the Huh7 sphere cells, respectively. TPGS-FA/NC (10、20 and 40 μg/mL) potently reduced the fraction of EpCAM+/CD133+ cells (Fig. 3a).

### TPGS-FA/NC inhibited hepatoma cell proliferation and colony formation

To test whether TPGS-FA/NC could sense the clonogenic assays, HCC cells (1 × 10^3^cells/well) were treated with or without TPGS-FA/NC in 6-well ultra-low attachment microplates and allowed to grow for 17 to 21 days. Importantly, the treatment inhibited Huh7 cell proliferation and also markedly reduced the number of colonies (Fig. 3b). Intriguingly, compared with control, TPGS-FA/NC showed no adverse effect in normal hepatic cell line L-02 by 40 μg/mL TPGS-FA/NC treatment (Fig. 3c).

### TPGS-FA/NC suppressed the AQP3 /CD133/STAT pathways

**Fig. 4:**
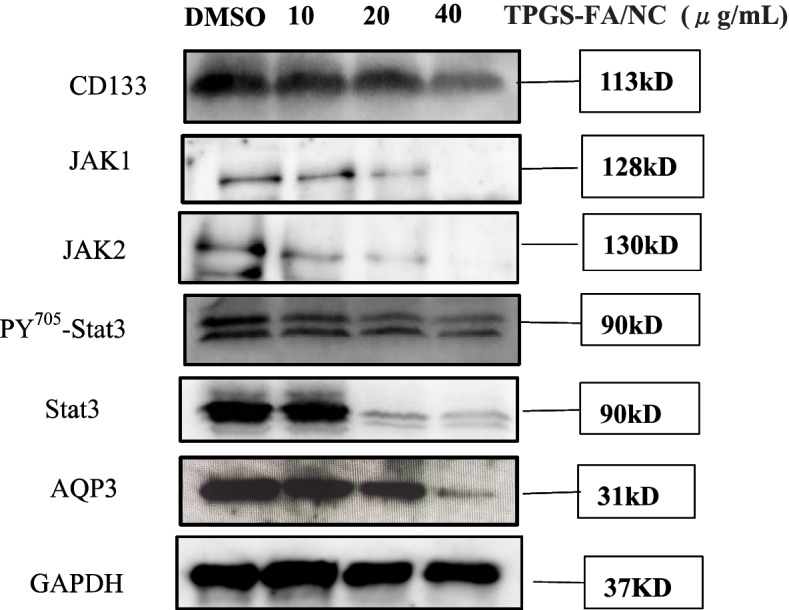
TPGS-FA/NC inhibits the AQP3/STAT3/CD133 pathway in Huh7 cells. Huh7 cells were treated with the indicated TPGS-FA/NC concentrations for 48 h. Western blotting was performed to determine the AQP3/STAT3/CD133 signaling-associated protein levels

**Fig. 5:**
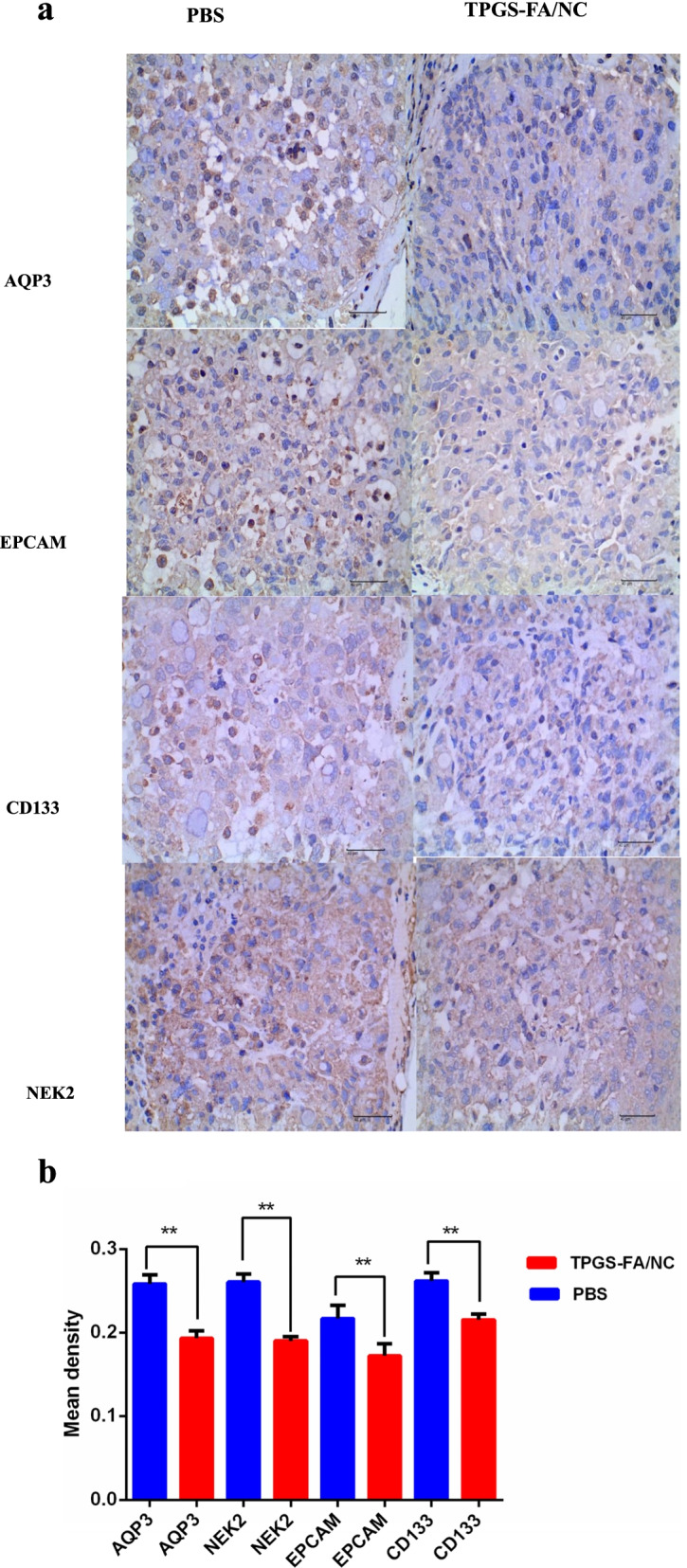
TPGS-FA/NC suppresses the protein levels in Huh7 xenograft specimens. **a, b** The protein of AQP3, NEK2,EPCAM, and CD133 (× 400, 40 μm) were mainly located in cytomembrane by immunohistochemical assay in 20 HCC mice bearing Huh7 xenograft specimens(**p < 0.01) *n* = 3. Mean ± SD. * *P* < 0.05 vs control. # *P* < 0.05 vs TPGS-FA/NC

Next, we set out to expore the mechanism of TPGS-FA/NC on AQP3 /CD133/STAT axis inhibittion. We found that TPGS-FA/NC reduced the protein expression levels of JAK1, JAK2, pY705-STAT3, STAT3 (Fig. 4). Furthermore, TPGS-FA/NC reduced the AQP3 protein expression, which suppressed the expression of activated STAT3 (pY705-STAT3) (Fig. 4). Then, in vivo experiment, we set out to test the CD133 and AQP3 expression levels in sections of nude mice subcutaneous tumors by IHC. Collectively, these results signified that TPGS-FA/NC downregulated AQP3 and CD133 protein levels (Fig. 5).

### TPGS-FA/NC impaired NEK2/CD133/EpCAM signaling of HCC

Next, the protein levels of NEk2,CD133 and EpCAM were determined in cells and nude nice treated with and without TPGS-FA/NC. TPGS-FA/NC successfully reduced protein expression levels of NEk2, CD133 and EpCAM in HCC. In vivo experiment, we tested the CD133 and AQP3 expression levels in sections of nude mice subcutaneous tumors by IHC. Together, these results suggest that TPGS-FA/NC downregulated NEk2, CD133 and EpCAM protein levels (Fig. 5).

### In vivo significant inhibition of tumor by TPGS-FA/NC nanoparticles

**Fig. 6:**
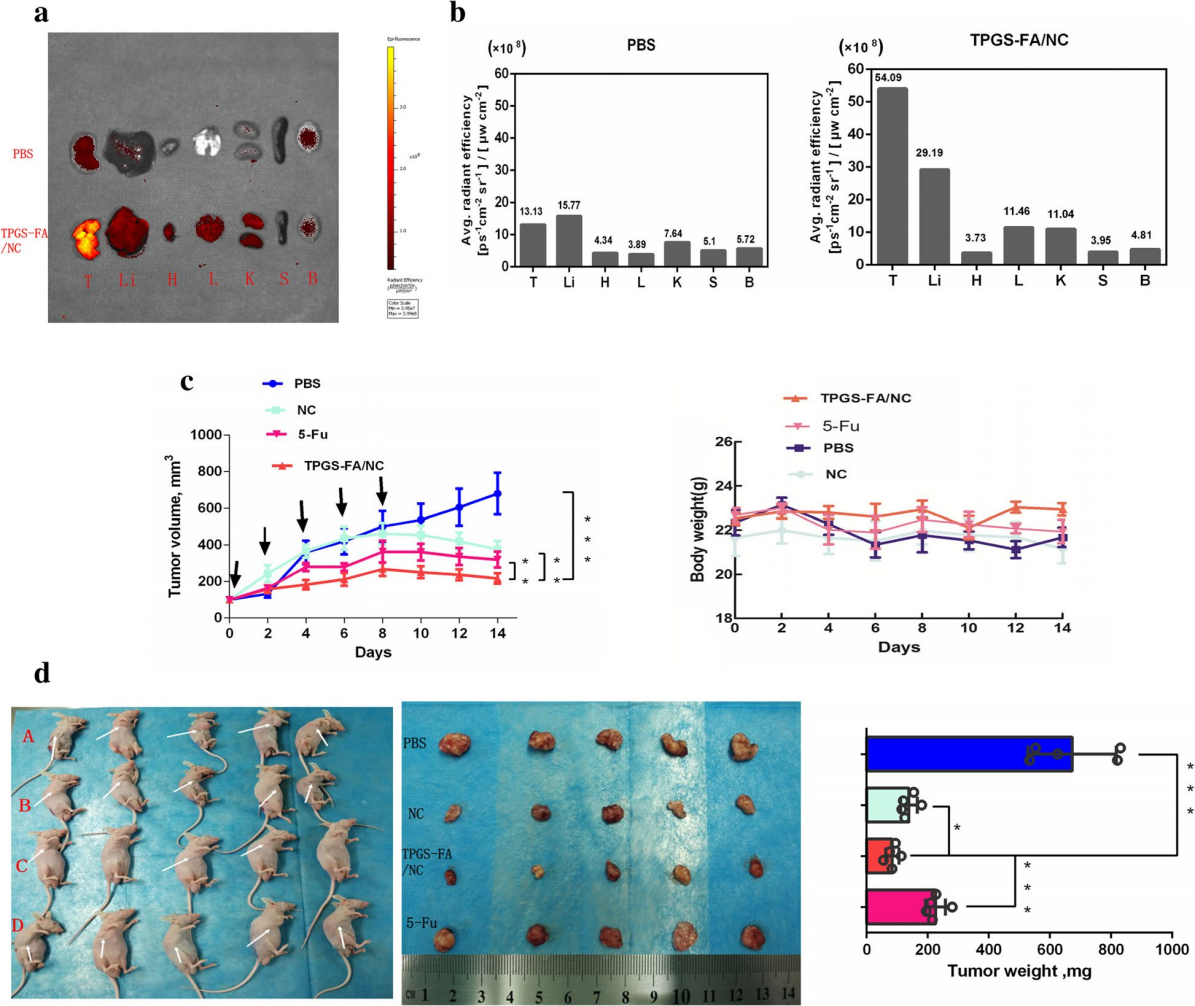
**a**. Representative organ images showing specific tumor targeting of rhodamine B isothiocyanate labeled TPGS-FA/NC nanoparticles 8 h post-injection into mice bearing Huh7 xenograft (T: tumor, Li: liver, H: heart, L: lung, K:kidney, S: spleen, and B:brain; Color scale: radiant efficiency, [p s^− 1^ cm^− 2^ sr^− 1^] [μWcm^− 2^]^− 1^). V **b**. Quantitative analysis of biodistribution in tumors and normal organs, quantified from the organ images. Intravenous treatment of nude mice bearing orthotopic Huh7 xenografts with TPGS-FA/NC nanoparticles (red) and control groups (turquoise: NC, fuchsia:5-Fu, blue: PBS) every other day for a total of five injections (4 mg kg − 1,NC per body weight, indicated by arrows). **c**. Mice body weight was monitored during the time course of treatments (*n* = 5 biologically independent animals, statistics was calculated by two-tailed unpaired t-test presented as mean ± SD, *p < 0.05, **p < 0.01, ***p < 0.001, *p* = 4.3 × 10^− 3^,3.4 × 10^− 3^ and 5.0 × 10^− 4^ comparing TPGS-FA/NC to NC,5-Fu and PBS, respectively). **d**. Representative images of liver cancer tumors harvested from mice after treatments *p < 0.05, **p < 0.01, ***p < 0.001; *p* = 0.01, 8 × 10 − 4,and 2 × 10 − 4 comparing TPGS-FA/NC to NC, 5-Fu, and PBS, respectively. Source data are provided as a Source Data file

**Fig. 7:**
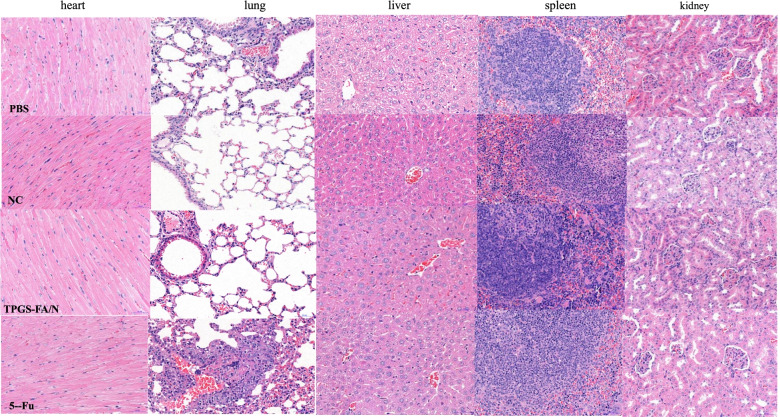
Representative H&E staining of HCC mice bearing Huh7 xenograft specimens

Thinking from a therapeutic notion, We then Tumor quantitative biodistribution and targeting of TPGS-FA/NC were assessed, which were injected through the tail vein in vivo. Those images of mice 8 h post-injection showed that the TPGS-FA/NC nanoparticles markedly accumulated in tumor, with low or no accumulation in brain, heart,spleen. (Fig. 6a). Quantitative analysis of the organ images showed strongly tumor accumulation. (Fig. 6b). After injecting with TPGS-FA/NC at a dose of 4 mgkg^− 1^ (NC per mouse weight) every 2 days for a total of five dosages, the results revealed a inhibitory capability in vivo as administration by tumor volumes, whereas control group (Fig. 6c). The specific tumor inhibition was further confirmed from the tumors harvested after 2-week post injections (Fig. 6d). Of note, the effects of those nanoparticles were biocompatible, suggesting no obvious organ toxicity over two-week post injections (Fig. 7).

## Discussion

Recently it has been shown that nitidine chloride (NC) inhibits the growth of many human cancer cells via induction of cell apoptosis [23]. In this study, we modify nitidine chloride to achieve a novel nitidine chloride nanoparticle using TPGS-FA carriers. TPGS(D-α-tocopheryl polyethylene glycol 1000 succinate) is a very safe biocom-patible and safe agent that can efficiently for use as a drug solubilizer [24–26]. Consistently, these results suggest no adverse effects of mice injected with TPGS-FA/NC. Importantly, this study provideda new target or method in treatment of hepatocellular carcinoma.

Interestingly, we observed TPGS-FA/NC significantly inhibited Huh7 cellular proliferation and colony formation. Importantly, normal hepatic cell line L-02 were not affected by 40 μg/mL TPGS-FA/NC treatment. Moreover, We examined a targeting effect of TPGS-FA/NC for hepatoma cells. Interestingly, it has been shown the use of the sphere culture technique and flow cytometry to enrich characterize hepatic CSCs [10, 11, 27]. Consistent with previous studies [11], Huh7 sphere cells exhibited CSC membrane biomarkers (EpCAM and CD133) including cell self-renewal capacities. In addition, we have shown that that the TPGS-FA/NC treatment markedly reduced the positive EpCAM/CD133 cell fraction as examined by FACS analysis, which suggested to be related with a suppressed self-renewal capability of these cancer stem-like cells. Meanwhile, we revealed that TPGS-FA/NC markedly reduced the numbers and sizes of the spheres. Moreover, TPGS-FA/NC (4 mg/kg) administration for 14d notably inhibited Huh7 xenograft tumor growth. Based on preliminary work, we reveals that the potential contribution of TPGS-FA/NC may serve as a promising drug in preventing and treating liver cancer.

AQP3/STAT3/CD133 pathway is a novel mechanism in the signal protein expression related to cell proliferation, transcription and survival [16]. AQP3/STAT3/CD133 signaling plays a major role during tumor progression in HCC [16]. Recently, AQP3 has been shown to express in various cancer cells including multiple cancer tissues from stomach, colon, and lung [28–30]. Meanwhile, Nek2 is reported to inhibit cancer cell proliferation and promote tumorigenesis and progression in HCC and colon cancer [31]. Consistent with these studies, we showed that TPGS-FA/NC suppressed the AQP3/STAT3/CD133 pathway and Nek2 expression in HCC cells, which was evidenced by reduced overexpression of AQP3 and STAT3, as well as downregulating the expression of CD133 through attenuating the stemness of CD133^+^ cells. Therefore, the downregulation of the AQP3/STAT3/CD133 pathway may be have contributed to the inhibitory effect of TPGS-FA/NC on HCC cells and hepatic CSCs.

## Conclusion

In conclusion, we have shown that TPGS-FA/NC is an effective inhibitor of HCC tumor growth with low toxicity. Furthermore, this study demonstrates that TPGS-FA/NC suppresses hepatoma cell proliferation and hepatic CSCs, as well as the AQP3/STAT3/CD133 axis and Nek2 expression in offering possible multiple mechanisms for its antitumor activity. Further clinical works are warranted to investigate the long-term effects of TPGS-FA/NC on tumor metastasis control and extending the patient’s survival .

## Supplementary Information

**Additional file 1.**

## Data Availability

All data generated or analyzed during the present study are included in this article. Supplementary data are present in the supplemental materials. Additional data related to this paper can be requested from the author (lidanni@gxmzu.edu.cn).
