# Supplementary material for: A novel nitidine chloride nanoparticle overcomes the stemness of CD133+EPCAM+ Huh7 hepatocellular carcinoma cells for liver cancer therapy
Source: BMC Pharmacol Toxicol. 2022 Jul 12;23:48. doi: 10.1186/s40360-022-00589-z (PMC9277916; doi:10.1186/s40360-022-00589-z)

Supplementary original western blot images

CD133

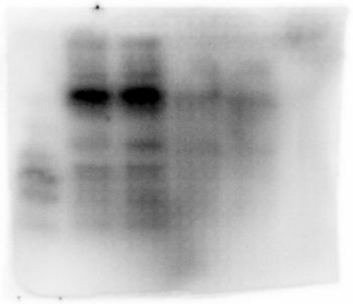

GAPDH

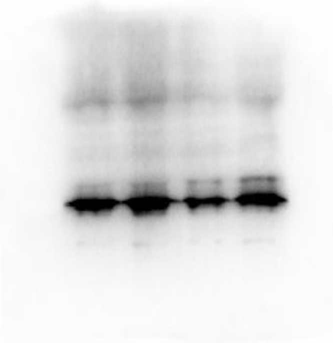

CD133

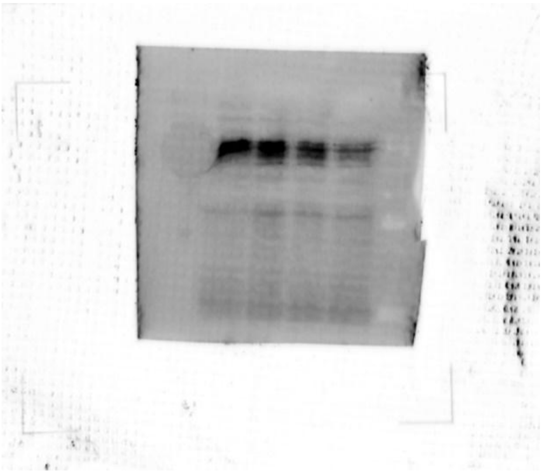

GAPDH

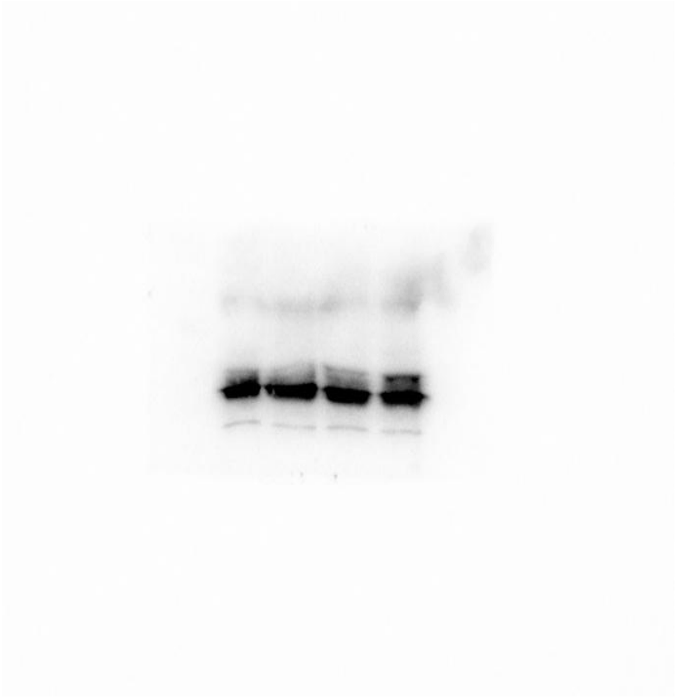

CD133

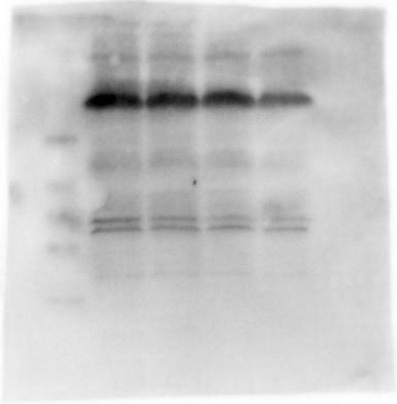

GAPDH

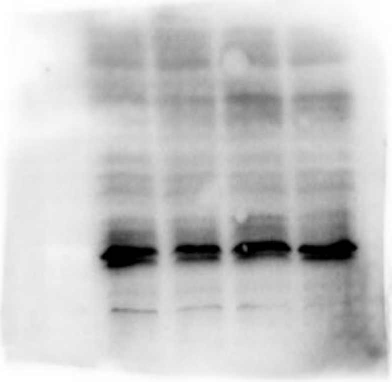

JAK1

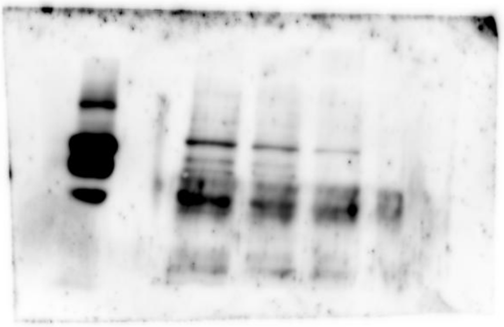

GAPDH

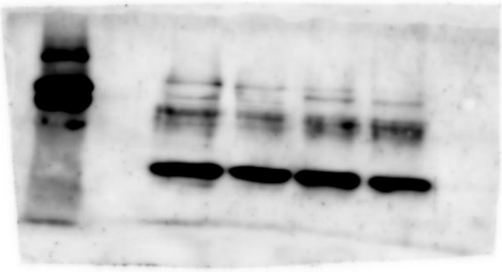

JAK1

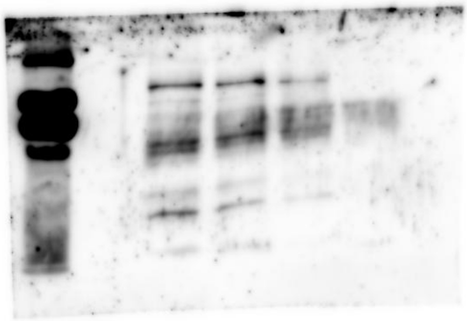

GAPDH

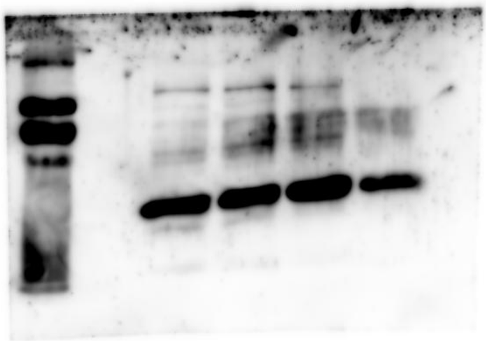

JAK1

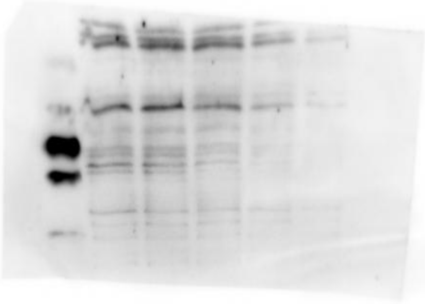

GAPDH

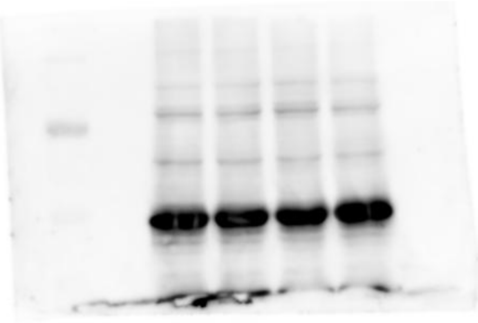

JAK2

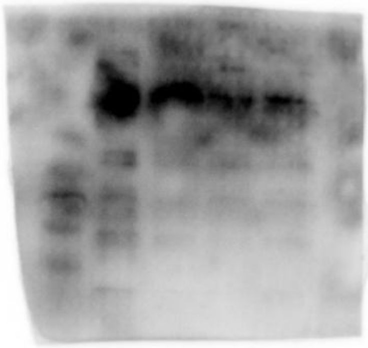

GAPDH

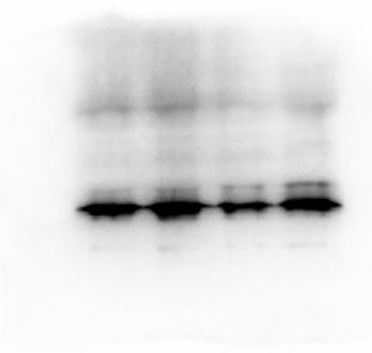

JAK2

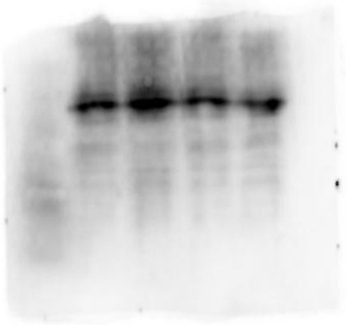

GAPDH

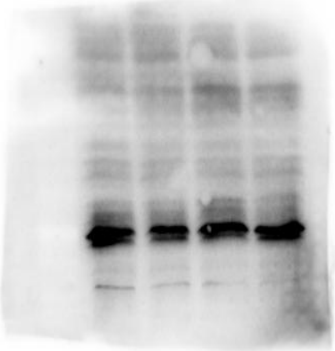

JAK2

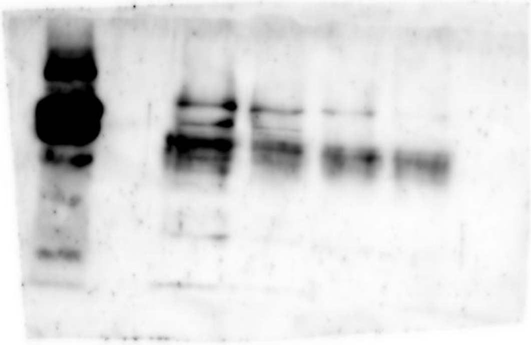

GAPDH

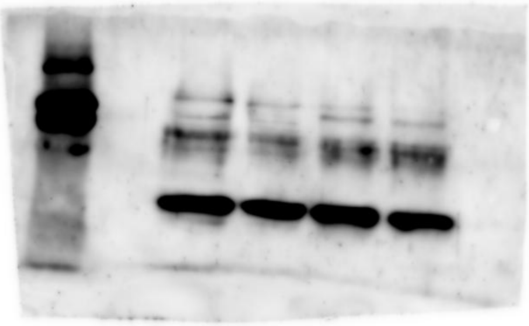

PY705 stat3

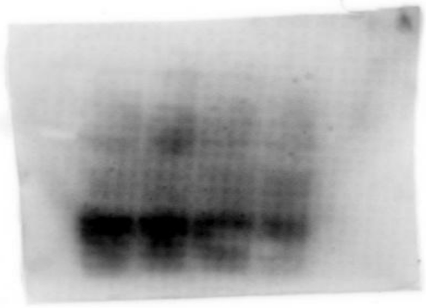

GAPDH

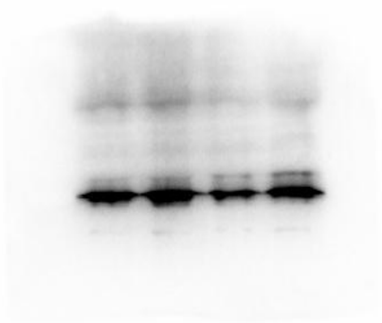

PY705 stat3

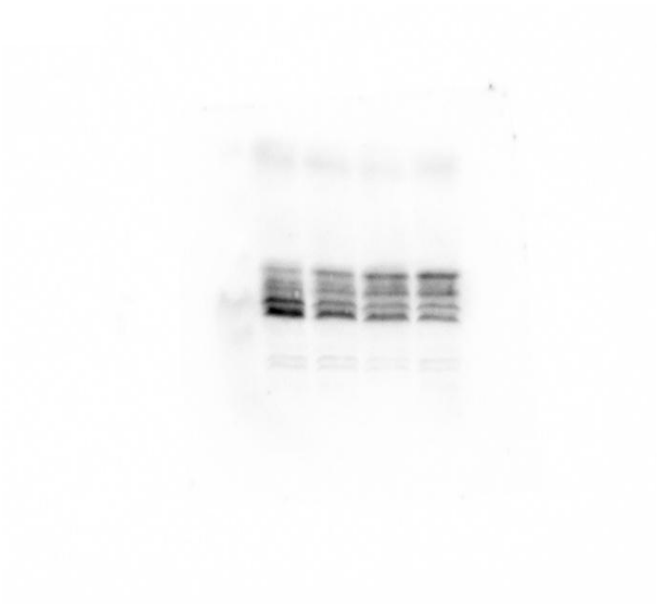

GAPDH

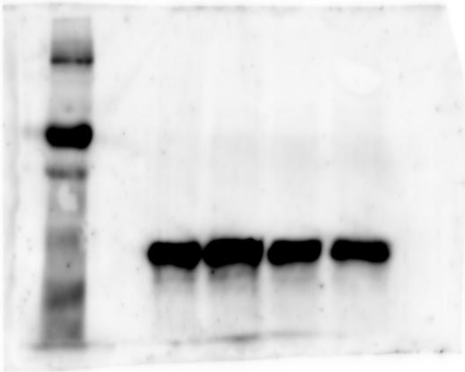

PY705 stat3

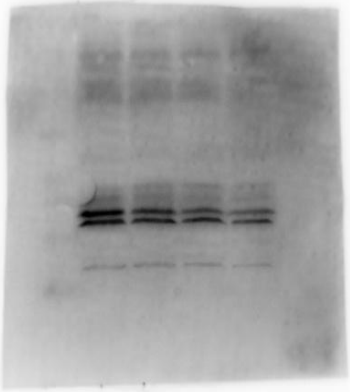

GAPDH

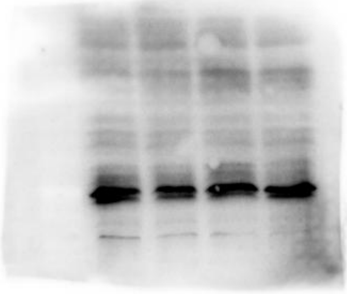

stat3

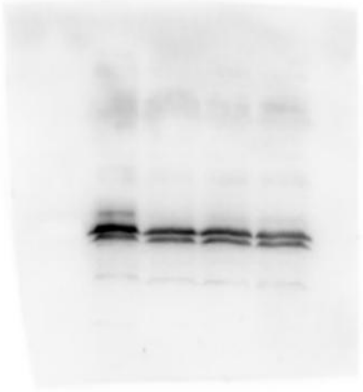

GAPDH

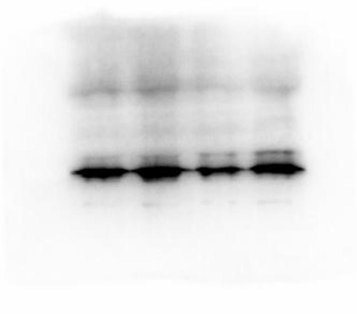

stat3

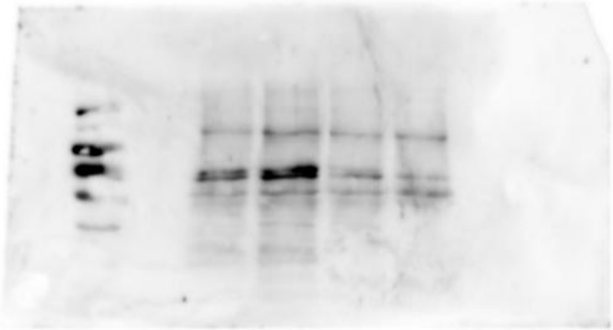

GAPDH

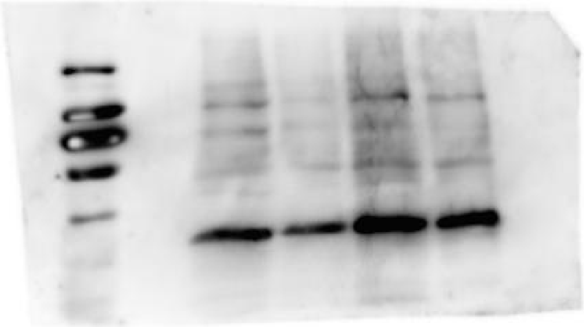

stat3

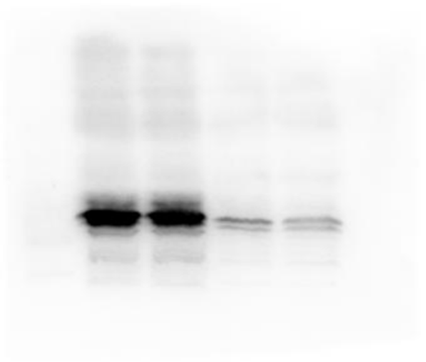

GAPDH

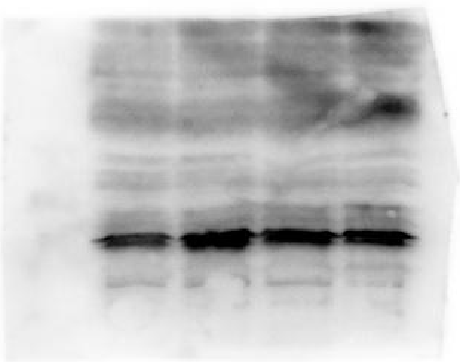

AQP3

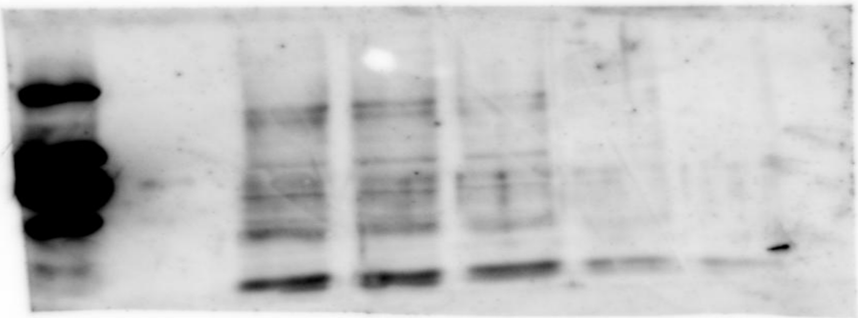

GAPDH

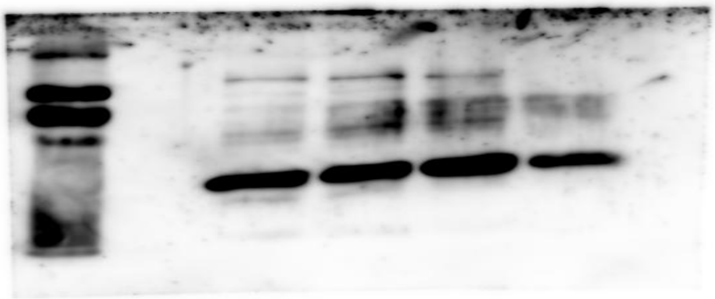

AQP3

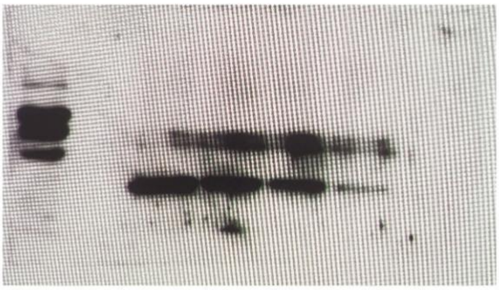

GAPDH

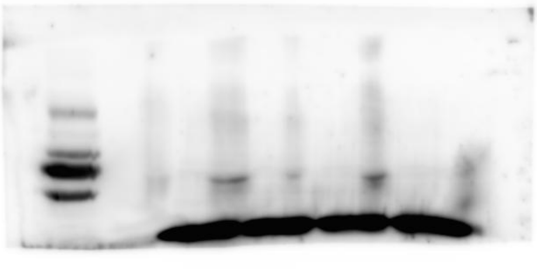

AQP3

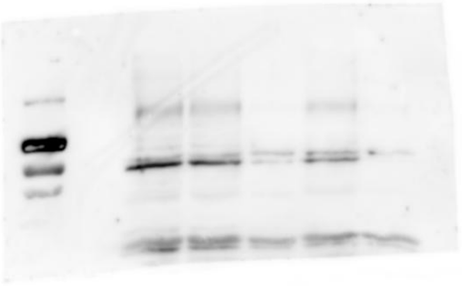

GAPDH

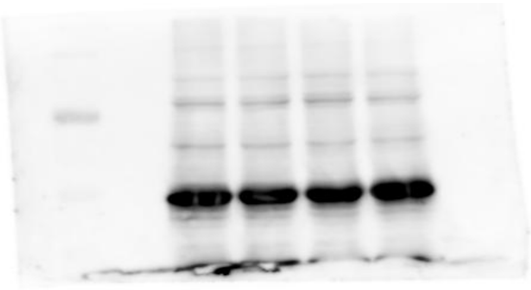

Supplement: Supplementary file 1 — Additional file 1. [file 40360_2022_589_MOESM1_ESM.zip › Supplementary original western blot images_20220705135117.pdf]
