# Supplementary material for: A novel nitidine chloride nanoparticle overcomes the stemness of CD133+EPCAM+ Huh7 hepatocellular carcinoma cells for liver cancer therapy
Source: BMC Pharmacol Toxicol. 2022 Jul 12;23:48. doi: 10.1186/s40360-022-00589-z (PMC9277916; doi:10.1186/s40360-022-00589-z)
Supplement: Supplementary file 1 — Additional file 1. [file 40360_2022_589_MOESM1_ESM.zip › Supplementary flow cytometry images.pdf]

Supplementary flow cytometry images

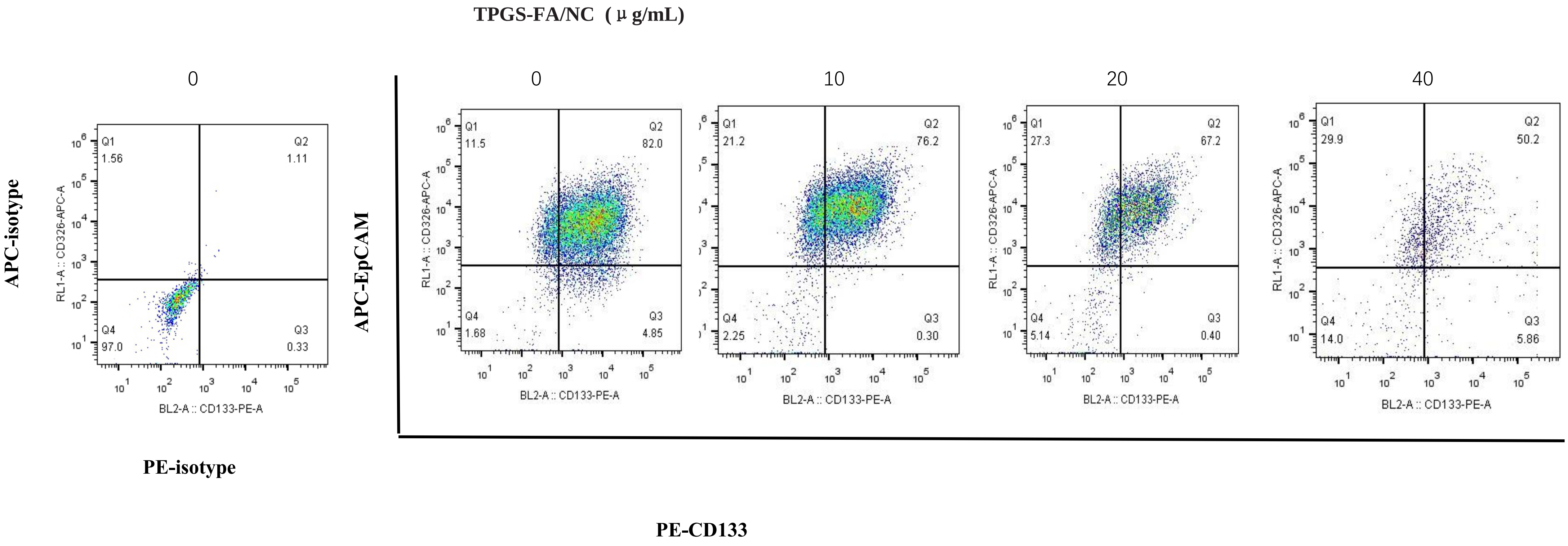

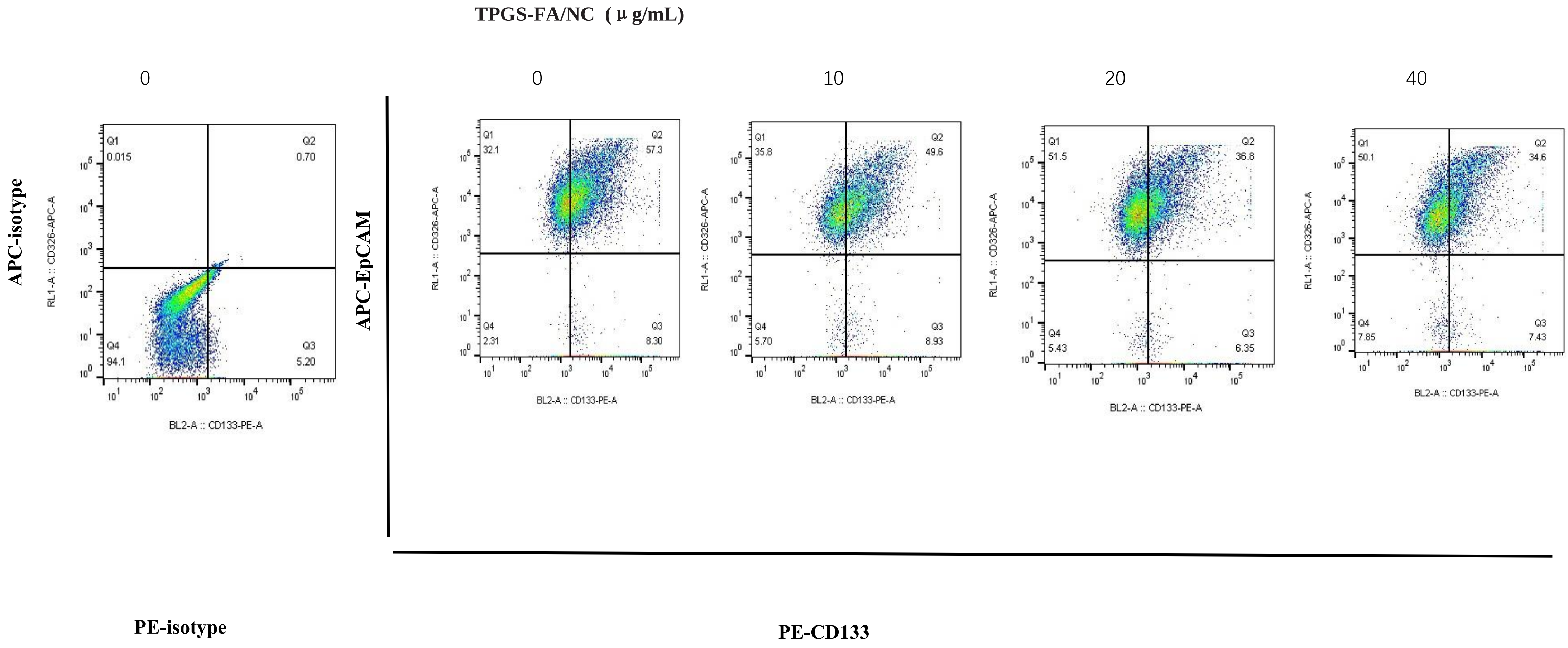
TPGS-FA/NC（μg/mL)
0
0
10
20
40
APC-isotype
APC-EpCAM
RL1-A :: CD326-APC-A
BL2-A :: CD133-PE-A
Q1 0.015
Q2 0.70
Q3 5.20
Q4 94.1
Q1 32.1
Q2 57.3
Q3 8.30
Q4 2.31
Q1 35.8
Q2 49.6
Q3 8.93
Q4 5.70
Q1 51.5
Q2 36.8
Q3 6.35
Q4 5.43
Q1 50.1
Q2 34.6
Q3 7.43
Q4 7.85
PE-isotype
PE-CD133

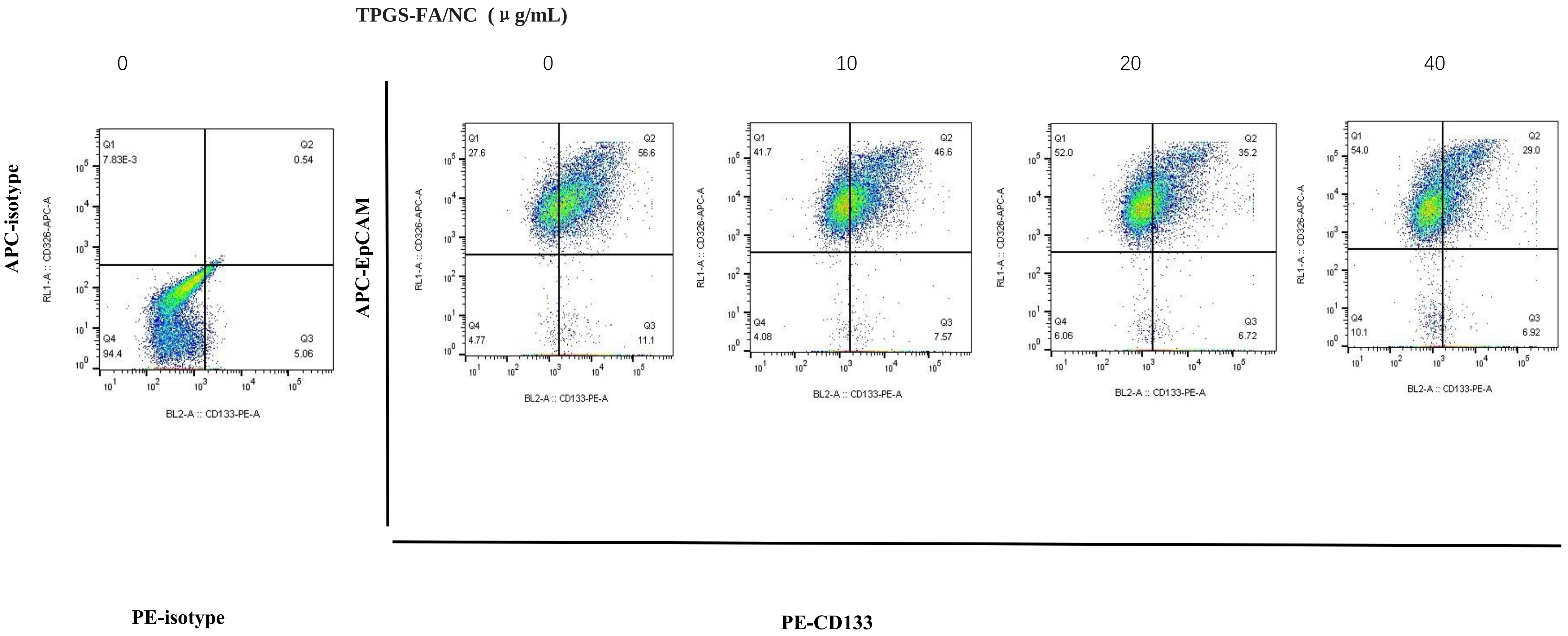
TPGS-FA/NC（μg/mL)
0
0
10
20
40
APC-isotype
APC-EpCAM
PE-isotype
PE-CD133
RL1-A :: CD326-APC-A
BL2-A :: CD133-PE-A
Q1 7.83E-3
Q2 0.54
Q4 94.4
Q3 5.06
Q1 27.6
Q2 56.6
Q4 4.77
Q3 11.1
Q1 41.7
Q2 46.6
Q4 4.08
Q3 7.57
Q1 52.0
Q2 35.2
Q4 6.06
Q3 6.72
Q1 54.0
Q2 29.0
Q4 10.1
Q3 6.92
