# Supplementary material for: A novel nitidine chloride nanoparticle overcomes the stemness of CD133+EPCAM+ Huh7 hepatocellular carcinoma cells for liver cancer therapy
Source: BMC Pharmacol Toxicol. 2022 Jul 12;23:48. doi: 10.1186/s40360-022-00589-z (PMC9277916; doi:10.1186/s40360-022-00589-z)

HL-7702[LO2] cells STR report

Method: An appropriate amount of HL-7702[LO2] cells (1×10^6) was used PureLink® Genomic DNA Mini Kit (USA Life K182001) to extract DNA, amplified by PowerPlex®18D system（USA Promega DC1802） kit, product detection was performed by ABI3500 Genetic Analyzer（USA Life3500）.

Experimental result:

1. The results of negative and positive control were correct.
2. The genotyping results of STR locus of HL-7702[LO2] cell line are shown in the following table.

Conclusion:

1. The genomic DNA of HL-7702[LO2] cell line is clear and the result of genotyping is good.
2. The results of STR typing showed that no cross contamination of human cells was found in the cell line of HL-7702[LO2] cell line.
3. The DNA typing of the cell line was 100.00% matched with the cell type in the CRC cell bank, and the cell line name was HL-7702[LO2].

Procell www.procell.com.cn

Appendix I: The genotyping results of STR locus of HL-7702[LO2] cell line

Marker	*Allele 1*	*Allele 2*
D3S1358	18	18
TH01	7	7
D21S11	27	28
D18S51	16	16
PentaE	7	17
D5S818	11	12
D13S317	13.3	13.3
D7S820	12	12
D16S539	9	10
CSF1PO	10	10
PentaD	8	15
AMEL	X	X
vWA	16	18
D8S1179	12	12
TPOX	12	12
FGA	18	21
D19S433	13	13
D2S1338	17	17
D1S1656	12	15
D6S1043	18	18
D12S391	20	25

Procell www.procell.com.cn

Figure 1: STR site database HL-7702[LO2] cell comparison information

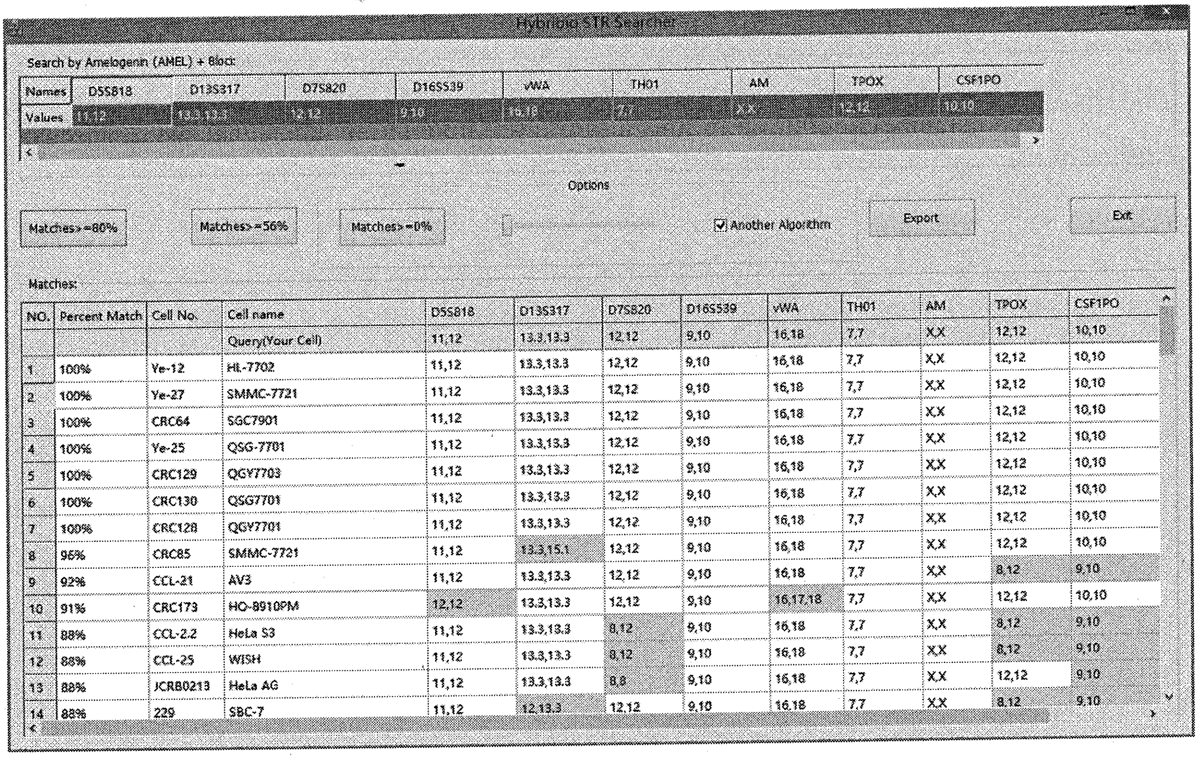

Names	D5S818	D13S317	D7S820	D16S539	vWA	TH01	AM	TPOX	CSF1PO
Values	11,12	13.3,13.3	12,12	9,10	16,18	7,7	X,X	12,12	10,10

NO.	Percent Match	Cell No.	Cell name	D5S818	D13S317	D7S820	D16S539	vWA	TH01	AM	TPOX	CSF1PO
			Query(Your Cell)	11,12	13.3,13.3	12,12	9,10	16,18	7,7	X,X	12,12	10,10
1	100%	Ye-12	HL-7702	11,12	13.3,13.3	12,12	9,10	16,18	7,7	X,X	12,12	10,10
2	100%	Ye-27	SMMC-7721	11,12	13.3,13.3	12,12	9,10	16,18	7,7	X,X	12,12	10,10
3	100%	CRC64	SGC7901	11,12	13.3,13.3	12,12	9,10	16,18	7,7	X,X	12,12	10,10
4	100%	Ye-25	QSG-7701	11,12	13.3,13.3	12,12	9,10	16,18	7,7	X,X	12,12	10,10
5	100%	CRC129	QGY7703	11,12	13.3,13.3	12,12	9,10	16,18	7,7	X,X	12,12	10,10
6	100%	CRC130	QSG7701	11,12	13.3,13.3	12,12	9,10	16,18	7,7	X,X	12,12	10,10
7	100%	CRC128	QGY7701	11,12	13.3,13.3	12,12	9,10	16,18	7,7	X,X	12,12	10,10
8	96%	CRC85	SMMC-7721	11,12	13.3,15.1	12,12	9,10	16,18	7,7	X,X	12,12	10,10
9	92%	CCL-21	AV3	11,12	13.3,13.3	12,12	9,10	16,18	7,7	X,X	8,12	9,10
10	91%	CRC173	HO-8910PM	12,12	13.3,13.3	12,12	9,10	16,17,18	7,7	X,X	12,12	10,10
11	88%	CCL-2.2	HeLa S3	11,12	13.3,13.3	8,12	9,10	16,18	7,7	X,X	8,12	9,10
12	88%	CCL-25	WISH	11,12	13.3,13.3	8,12	9,10	16,18	7,7	X,X	8,12	9,10
13	88%	JCRB0213	HeLa AG	11,12	13.3,13.3	8,8	9,10	16,18	7,7	X,X	12,12	9,10
14	88%	229	SBC-7	11,12	12,13.3	12,12	9,10	16,18	7,7	X,X	8,12	9,10

Appendix II: The genotyping results of STR locus of HL-7702[LO2] cell line

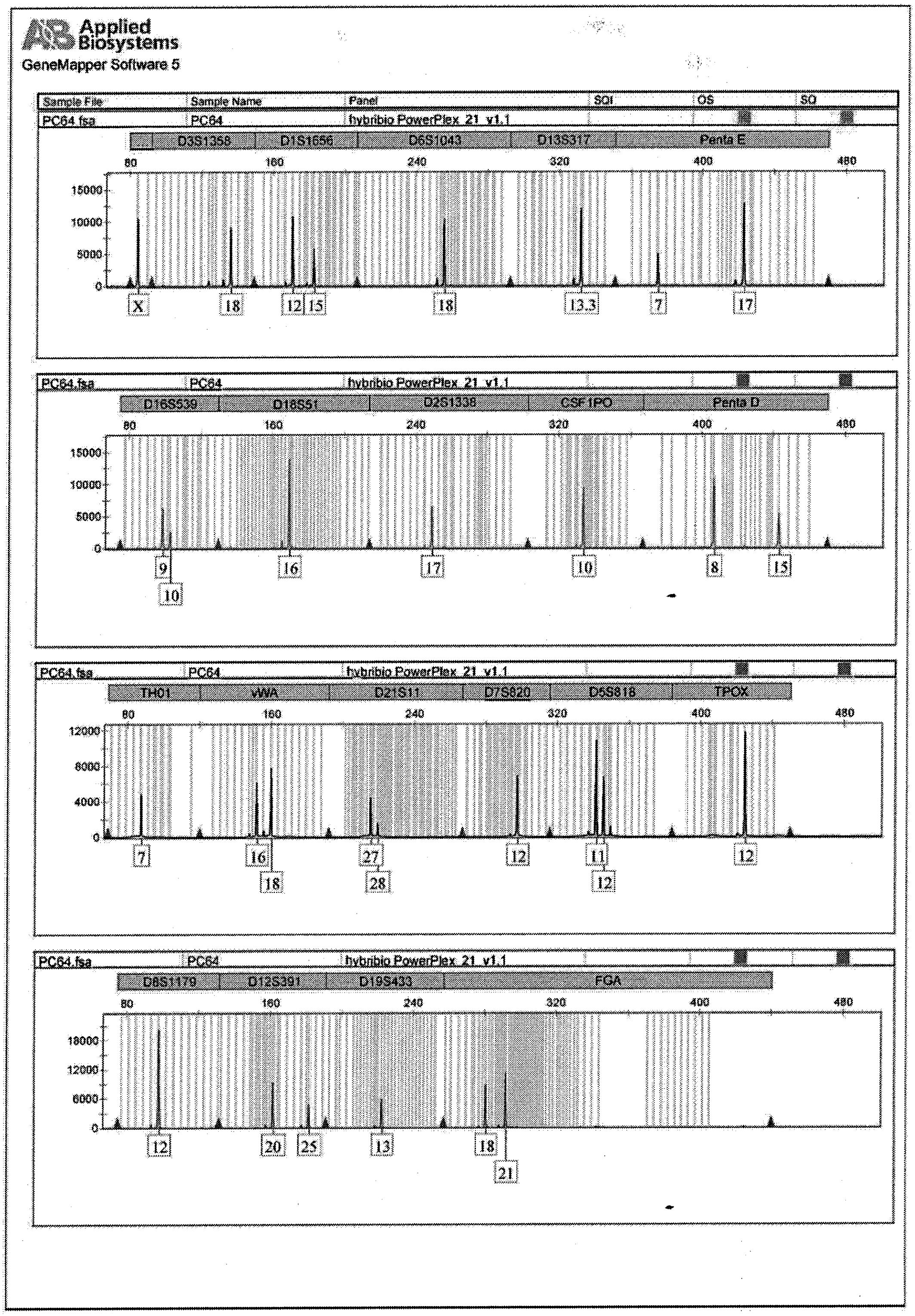

Supplement: Supplementary file 1 — Additional file 1. [file 40360_2022_589_MOESM1_ESM.zip › Supplementary materia 2.pdf]
